# Combined Model of Intrinsic and Extrinsic Variability for Computational Network Design with Application to Synthetic Biology

**DOI:** 10.1371/journal.pcbi.1002960

**Published:** 2013-03-28

**Authors:** Tina Toni, Bruce Tidor

**Affiliations:** 1 Department of Biological Engineering, Massachusetts Institute of Technology, Cambridge, Massachusetts, United States of America; 2 Computer Science and Artificial Intelligence Laboratory, Massachusetts Institute of Technology, Cambridge, Massachusetts, United States of America; 3 Department of Electrical Engineering and Computer Science, Massachusetts Institute of Technology, Cambridge, Massachusetts, United States of America; Johns Hopkins University, United States of America

## Abstract

Biological systems are inherently variable, with their dynamics influenced by intrinsic and extrinsic sources. These systems are often only partially characterized, with large uncertainties about specific sources of extrinsic variability and biochemical properties. Moreover, it is not yet well understood how different sources of variability combine and affect biological systems in concert. To successfully design biomedical therapies or synthetic circuits with robust performance, it is crucial to account for uncertainty and effects of variability. Here we introduce an efficient modeling and simulation framework to study systems that are simultaneously subject to multiple sources of variability, and apply it to make design decisions on small genetic networks that play a role of basic design elements of synthetic circuits. Specifically, the framework was used to explore the effect of transcriptional and post-transcriptional autoregulation on fluctuations in protein expression in simple genetic networks. We found that autoregulation could either suppress or increase the output variability, depending on specific noise sources and network parameters. We showed that transcriptional autoregulation was more successful than post-transcriptional in suppressing variability across a wide range of intrinsic and extrinsic magnitudes and sources. We derived the following design principles to guide the design of circuits that best suppress variability: (i) high protein cooperativity and low miRNA cooperativity, (ii) imperfect complementarity between miRNA and mRNA was preferred to perfect complementarity, and (iii) correlated expression of mRNA and miRNA – for example, on the same transcript – was best for suppression of protein variability. Results further showed that correlations in kinetic parameters between cells affected the ability to suppress variability, and that variability in transient states did not necessarily follow the same principles as variability in the steady state. Our model and findings provide a general framework to guide design principles in synthetic biology.

## Author Summary

Variability is inherent in biological systems, and in order to understand them, we need to be able to model different sources of variability. Systems have evolved to harness and control the variability, and more recently, synthetic biologists are trying to learn how to control variability in engineered biological systems. Several sources of variability exist; they arise due to stochastic expression of genes, which is most pronounced when numbers of mRNA and protein molecules are low, as well as due to differences between individual cells. Here we propose a modeling framework that combines different sources of biological variability. Furthermore, current research seeks to control biological variability though robust design of synthetic biological circuits, for example for use in therapies and other biomedical or biotechnological applications. Here we apply our framework to guide design of synthetic circuits that use transcriptional and post-transcriptional regulation to suppress variability in the output protein of interest. We find that certain properties and network designs are better than others in their ability to control variability, and here we report on the design guidelines to aid synthetic circuit design to suppress variability, in spite of our uncertain knowledge of parameters.

## Introduction

Biological systems are complex, inherently noisy and only partially understood [1]–[5]. Systems and synthetic biologists are striving to better understand these systems, as well as to discover generally applicable principles for controlling them in biomedical and biotechnological applications. For example, a branch of systems biology studies how to best interfere with variable and under-characterized signaling pathways to identify novel drug targets [6]–[8]. In clinical pharmacology, decisions need to be made by using uncertain models of drug effects on the human body across populations and should ideally be robust to patient-to-patient differences [9], [10]. Synthetic biologists strive to build synthetic circuits that perform desired functions across a population of cells, despite their noisy nature, cell-to-cell variability, and changing environments [11], [12].

We are faced with a challenge of how to best represent, simulate, analyze, and carry out design for noisy systems with under-characterized biochemical properties, which are often represented as uncertain parameters. We propose a modeling and simulation framework as a tool to aid in meeting these challenges. In this manuscript we specifically focus on making design decisions for synthetic genetic networks, but the modeling technique is sufficiently general to be applicable to a wide range of problems in biology, biological engineering, and medicine. The framework accounts for different sources of variability and is computationally efficient, so that it allows screening across broad parameter ranges.

Intrinsic variability (or intrinsic stochasticity) is a relatively well understood aspect of biological models. It arises from the probabilistic nature of the timing of collision events between reacting biological molecules, and its effect is most pronounced when the number of molecules in the system is small. Traditionally intrinsic variability is modeled by a stochastic master equation, which is the foundation for modeling stochastic dynamics in most physical, chemical, and biological phenomena [13]. Unfortunately, its analytic solution can only be found for a few trivial models, and a good alternative for studying stochastic models is the exact simulation framework of Gillespie [14]. However, to obtain a distribution resulting from the intrinsic variability, many trajectories of the Gillespie algorithm need to be simulated, which can be computationally expensive. A practical alternative is to use analytically tractable approximation schemes [15], [16]. In this manuscript we approximate stochastic dynamics by van Kampen's 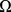-expansion [13] (also called the linear noise approximation or perturbation expansion; see Methods), for its computational efficiency and analytic form. The 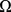-expansion separates the macroscopic dynamics from the fluctuations around it, describing each of these parts by a set of ordinary differential equations (ODEs). This model allows for efficient propagation of the first two moments of the intrinsic noise distribution through time using deterministic equations for mean, variances, and covariances. The model very accurately approximates stochastic dynamics for medium and large molecular numbers and when variability is small compared to the mean number of molecules, but can lose on accuracy when numbers of molecules are very small and the relative fluctuation size increases [17]–[19]; here we check using Gillespie simulations that the 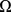-expansion distributions are accurate. Analytic studies of the accuracy of the 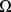-expansion and comparison with other traditional approaches for modeling intrinsic noise such as Fokker Planck equation and the chemical Langevin equation can be found in [17], [18], [20] and a summary on validity of the 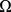-expansion in the Supplementary material of [19]. Despite this caveat, and mainly due to its efficiency and possibility of analytic study, the 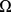-expansion has played an important role in advancing the understanding of intrinsic variability [3], [18], [21]–[24]. The 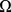-expansion can most conservatively be applied to models with a single steady state — in this manuscript we will only consider such models — but with certain limitations or modifications it can also be applied to multimodal and oscillatory models [22], [25]–[27].

Significant effort has been invested in modeling intrinsic variability in systems and synthetic biology, although it has been shown that extrinsic variability generally dominates, especially in eukaryotic systems [2], [28]. Extrinsic variability arises due to varying components upstream of the system of interest; these components affect the system, varying stochastically in time themselves, and might be present in different amounts in cells due to differences such as size and stage of the cell cycle [2], [3], [29]. For example, the numbers of ribosomes and the numbers of RNA polymerases vary in time and between cells. Another source of extrinsic variability is cell-to-cell variability of the gene copy number, which is common in synthetic biology applications when genes are delivered into cells by plasmid transfection, after which different numbers of plasmids are taken up by different cells. As a result of such sources of variability, single cells within a population possess distinct quantitative dynamic behaviors.

As yet, there is no commonly accepted framework for modeling extrinsic variability. Despite several strong mathematical and theoretical studies of intrinsic and extrinsic variability [2], [3], [30], [31], computational modeling efforts that combine intrinsic and extrinsic variability are still rare. Shahrezaei *et al.* [32] proposed an extension to the Gillespie approach that includes kinetic parameter perturbations representing extrinsic variability; the downside of this method is that it is extremely costly. Scott *et al.* [26] proposed a more efficient, approximate model for steady-state extrinsic variability that can account for variations of one parameter at a time. Zechner *et al.* [33] used low-order moments through the moment closure approach to approximate intrinsic and extrinsic distributions; this approach requires analytic derivation of a new model structure for each additional extrinsic factor. Hallen *et al.* [34] proposed a non-mechanistic method of modeling extrinsic variability by perturbing the steady-state intrinsic noise distribution, but without any mechanistic assumption regarding the sources of extrinsic variability. Singh *et al.* [35] model extrinsic variability by adding noisy exogenous signals to an intrinsic stochastic model.

**Figure 1:**
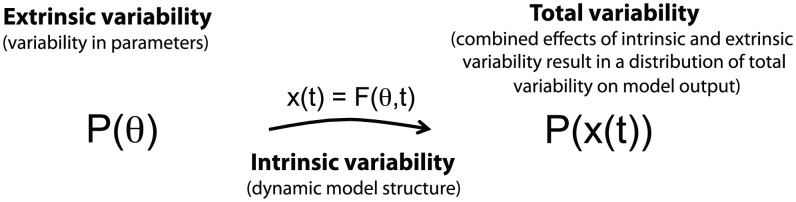
Framework overview. The framework combines intrinsic and extrinsic sources of variability and computes the total variability in model output. Extrinsic variability enters the model through variability in kinetic parameters and initial conditions. Intrinsic stochasticity is accounted for in the choice of a dynamical system; the master equation is the standard way of representing intrinsic variability, and due to its intractability we use an approximation of the master equation known as the 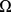-expansion.

Here we model extrinsic variability by introducing variability in model parameters and initial conditions; rather than considering them as point values, we consider them as distributions (Figures 1, S1). We propagate these distributions through a model to simulate model output distributions resulting from extrinsic variability. For computational convenience we work with normally distributed parameters, 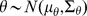 (for simplicity 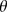 denotes a vector of all parameters and initial conditions). To simulate propagation of extrinsic variability through the model, we use the Unscented Transform (UT). The UT efficiently maps the first two moments of the variability distribution in the parameter space onto the first two moments of variability distribution in the output. Estimates of the mean and covariance matrix obtained by the UT are accurate to second order in the Taylor series expansion for any nonlinear function, which makes the algorithm very appealing for propagating distributions through nonlinear functions [36]. Nonlinearity is propagated through simulating the nonlinear function for a chosen set of parameters (called sigma-points, see Methods) and reconstructing the output distribution from these individual simulations. This can capture nonlinearity such as a shifts in a mean, for example, when extrinsic variability increases.

Despite knowing that extrinsic variability contributes significantly to the total variability, little is known about its sources [37]. This creates a major caveat that arises when attempting to make informed design decisions. The second ubiquitous caveat on the way to building predictive models of stochastic systems is that kinetic parameters are often unknown. Here we are motivated by a question of how to make robust design choices, given these uncertainties and limited knowledge of extrinsic variability.

In this paper we use our method to study variability in simple gene regulatory networks; such networks are a basis for transcription-based synthetic circuits. We first introduce a combined intrinsic and extrinsic modeling approach and derive the expression for the total variability. The framework is then used to explore the effect of self-repression on protein variability in simple genetic networks that are simultaneously under different sources of noise. We are interested in details related to how self-repression can be used as an element in synthetic circuits to reduce variability. We consider two types of self-repression, on a transcriptional and on a post-transcriptional level, and ask which is more successful in reducing protein variability. We are further interested in specific design principles that help optimally achieve the aim of noise suppression.

## Results

### Combined model of intrinsic and extrinsic variability, and derivation of total variability

**Figure 2:**
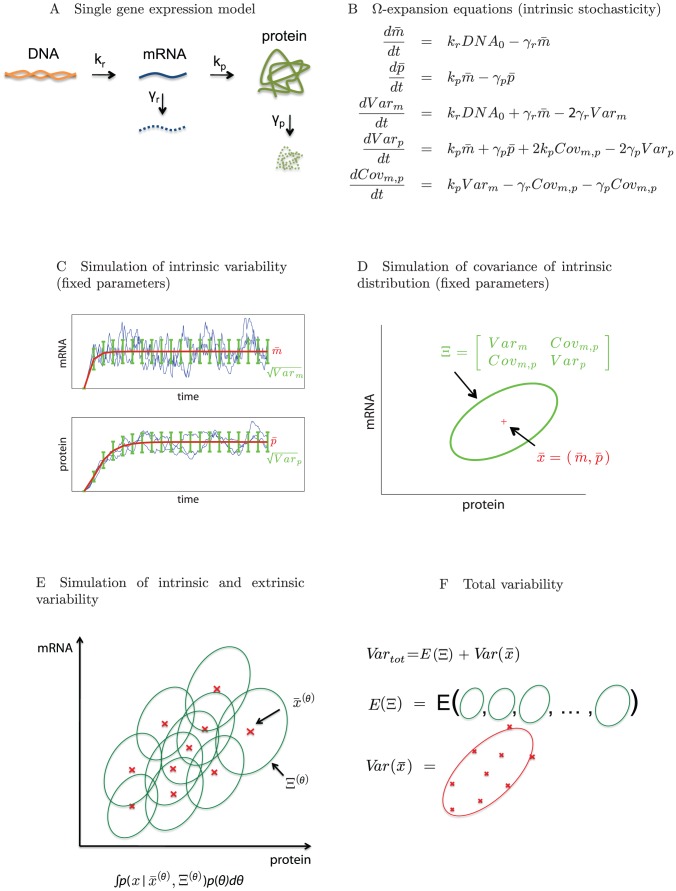
Framework illustrated on a single gene expression model. (A) Single gene expression model. (B) 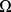-expansion equations (intrinsic stochasticity). (C) Simulation of intrinsic variability (fixed parameters). (D) Simulation of covariance of intrinsic distribution (fixed parameters). (E) Simulation of intrinsic and extrinsic variability. (F) Total variability.

The central methodological advance of this manuscript is a method and efficient framework to model total variability as a combination of intrinsic and extrinsic sources. Here we formalize the framework (overview in Figure 1) and illustrate it on a simple model of transcription and translation of a single gene, with species mRNA, 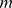, and protein, 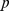 (Figure 2A) [38]. The parameters of the model are transcription rate, 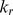, translation rate, 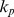, and mRNA and protein degradation rates, 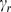 and 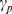, respectively. 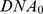 is the initial condition representing the copy number of genes encoding protein 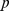. We denote the vector of parameters and initial conditions by 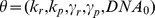.

Here we represent intrinsic variability through the 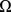-expansion, which provides a set of ordinary differential equations for the concentration means that are identical to a traditional ODE model without variability, plus an additional set of differential equations that describe time derivatives of the individual variances and covariances (Figure 2B). All equations together approximate the time evolution of the intrinsic noise distribution. Note that the ODEs for the means do not depend on the variances and covariances but that the ODEs for the variances and covariances depend on means, variances, and covariances. Note also that ODEs for propagating the variance and covariance do not introduce any additional rate constant parameters beyond those needed to propagate the concentration means, but that initial values for variance and covariance need to be introduced in order to integrate their differential equations. We express this as a system of ordinary differential equations
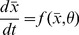
(1)

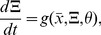
(2)where 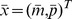 is a vector representing the mean numbers of mRNA and protein and 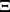 is the symmetric covariance matrix
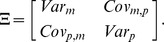



We simulated intrinsic variability in single gene expression using the 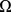-expansion model. Time-course trajectories of mean number of mRNA and protein (red) and their variances (green) are shown in Figure 2C. The Figure also shows individual trajectories from stochastic simulation runs (blue) using the Gillespie algorithm (Methods) [14], which are consistent with the 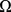-expansion simulations. A more detailed comparison shows that the distributions from the 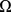-expansion and the stochastic Gillespie simulations are nearly identical (Figure S2). The multivariate Gaussian distribution 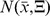 is depicted by an ellipse with mean 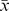 whose axes' directions are determined by eigenvectors and their sizes by eigenvalues of the covariance matrix 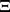 (Figure 2D, see Methods for further details). This represents variability in mRNA and protein counts due to intrinsic noise only.

We next introduced extrinsic variability into the model, by introducing parameters that follow a distribution with a probability density function 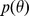, rather than parameters fixed to a particular value. Operationally, the combined distribution is a superposition of intrinsic distributions for different parameter realizations sampled from the underlying parameter probability distribution, 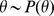 (Figure 2E). The resulting output distribution represents the *total variability* (i.e., variability resulting from intrinsic as well as extrinsic sources). Mathematically we represent this distribution with a mixture model with probability density function
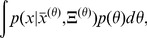
(3)where 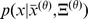 is a probability density of the intrinsic noise distribution, in our specific case a normal distribution with mean 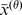 and a covariance matrix 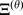 resulting from the 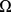-expansion model with parameters 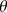. For single gene expression, the mixture model reads
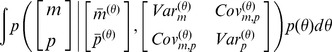
and its steady state is schematically depicted in Figure 2E.

This framework allows us to calculate the mean and variance of the total variability distribution; the mean of a random variable 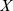 drawn from the mixture model (3) was calculated as
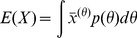
(4)and by using the law of total variance [39] we obtained the covariance matrix [30]

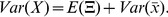
(5)


This equation shows that the total variability is decomposable into two parts: variability due to intrinsic stochasticity, 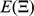, and variability due to extrinsic sources, 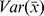 (Figure 2F).

So far we have not restricted ourselves to any specific form of the parameter distribution 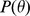; in order to obtain a combined model and calculate the total variability, we generally need to exhaustively draw samples from 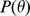 and simulate the 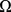-expansion model for each sampled parameter. However, for normally distributed parameters, 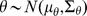, we can take a considerably more efficient approach, the Unscented Transform (UT, see Methods). The UT selects a small collection of representative control points in the parameter space (called sigma points), propagates them through the 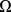-expansion simulation model to obtain the output instance for each, and then reconstructs the first two moments of the output distribution (which is equivalent to the above mixture model distribution) by appropriate weighting of the sigma points. From the output distribution, the total mean and the total variance are easily computed. See Text S1 for further details and an example for the single gene expression model.

We checked that our novel combined simulation framework accurately approximates the exact total protein and mRNA distributions resulting from Gillespie simulation. We first introduced extrinsic variability into one parameter only, and then into four parameters. In the first case, approximate distributions resulting from the novel framework fit very well to exact distributions in both steady and transient states (Figure S3). In the second example with extrinsic variability in four parameters, the approximation was slightly worse; this is becasue the total variability distribution was not Gaussian, but skewed, and perhaps also to inexactness in the 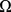-expansion (Figure S4). However, our approximation approximated well the distribution up to the second moment, i.e. the mean and the variance.

**Figure 3:**
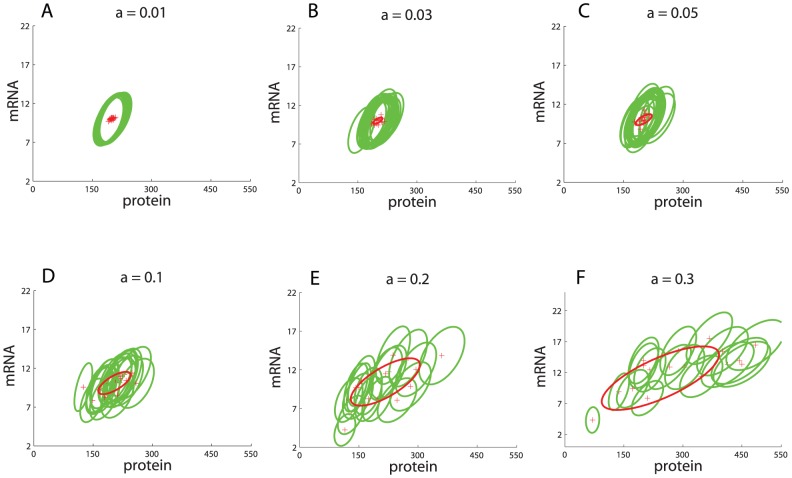
Distributions of the total variability. Distributions of the total variability result from the combined effects of intrinsic stochasticity and extrinsic variability due to parameter fluctuations in time and across cells. The amount of parameter variability is determined by Eqn. (13) in a diagonal covariance matrix 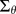. Variability in parameters is gradually increased: (A) 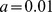, (B) 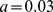, (C) 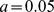, (D) 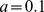, (E) 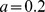, and (F) 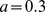.

Figure 3 shows examples of interplay between intrinsic and extrinsic variability for distinct amounts of extrinsic variability. In this example we considered parameters to be independent (i.e., the covariance matrix 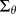 had zero for all off-diagonal entries), and all parameters were varied with the same coefficient of variation 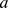 (see Methods). When the variability in parameters was low, intrinsic variability was dominant in the system (Figure 3A); this can be seen from the sizes of the green ellipses (the “average” of the green ellipses corresponds to 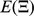 in Eq. 5), which are much larger than the size of the red ellipse (corresponding to 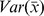). On the other hand, for high parameter variability the extrinsic variability became dominant (Figure 3F; red ellipse is larger than the green ones). For intermediate parameter variability, both intrinsic and extrinsic sources contributed comparably to the overall variability. Notice also that with increasing extrinsic variability, the intrinsic components changed their size and shape; this corresponds to the observation that intrinsic variability can change with kinetic parameters [40]. The total variability is the combination of red and averaged green ellipses; with increasing extrinsic variability, the variability of the output increased. High extrinsic variability has also shifted the mean; for example protein mean has increased when extrinsic variability was increased (this can be seen by comparing the x-component of the red ellipse mean on Figures 3A and 3F, where the mean of the red ellipse represents the mean of the output – combined intrinsic and extrinsic noise – distribution).

In the remainder of this paper we report results obtained by using this framework to study how different topologies of self-repressing genetic networks affect the total variability in proteins.

### The role of transcriptional and post-transcriptional autoregulation in reducing variability

**Figure 4:**
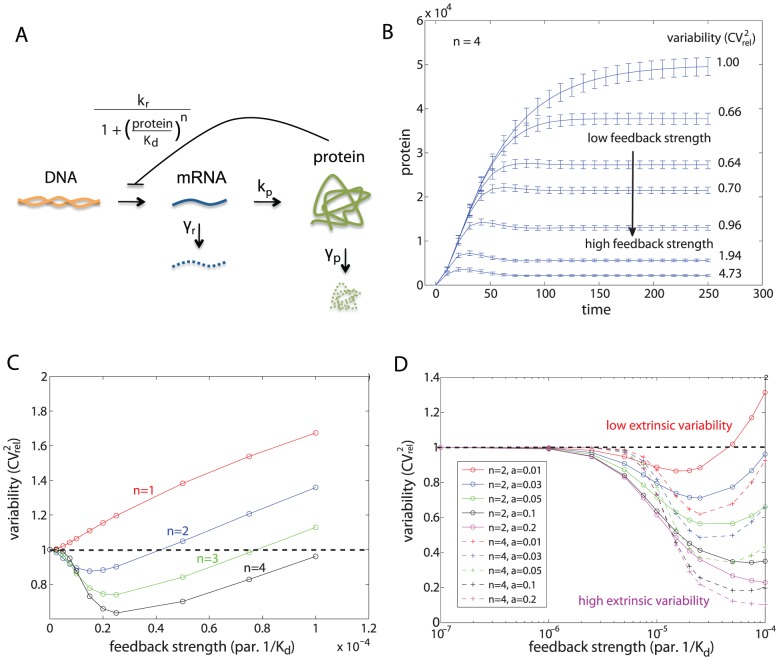
Results for the single gene expression model. (A) Single gene expression model with transcriptional self-repression. (B) Examples of protein distribution dynamics under no feedback (top-most) and under negative feedback. Increased feedback strength (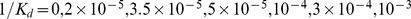 top to bottom) resulted in lower means and variances. Reported are the measures of variability calculated for the steady-state distribution with Hill coefficient 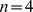. (C) Effect of self-repression on variability when intrinsic stochasticity is the only source of variability, for Hill coefficient values of 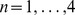. (D) Effect of self-repression on the system with both intrinsic and extrinsic variability. Extrinsic variability is introduced into the transcription rate. Extrinsic variability in parameters (eqn. (13)): 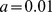 (red), 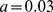 (blue), 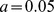 (green), 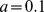 (black), 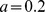 (magenta) with Hill coefficient 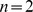 (full line; o), 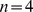 (dashed line; +).

**Figure 5:**
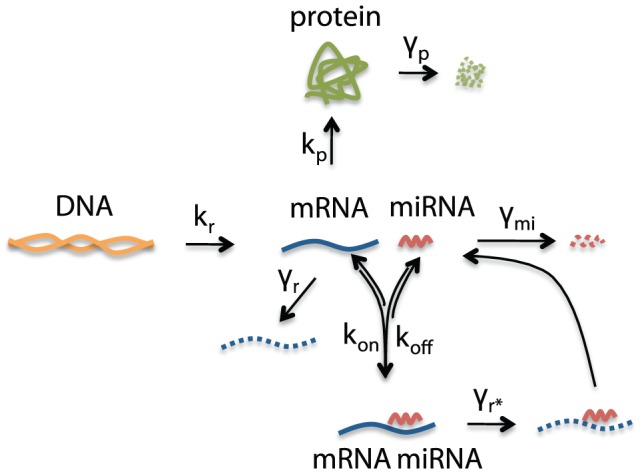
Single gene expression model with post-transcriptional self-repression.

Negative feedback and self-repression are recurrent motifs observed in gene regulatory networks. Their potential role in suppressing variability has identified them as promising design elements in synthetic biology [32], [41]–[48]. Here we studied the effects of self-repression on variability of the output protein in a single gene model, while taking into account both intrinsic and extrinsic sources of variability. We compared a case of negative autoregulation on a transcriptional level with a case of post-transcriptional regulation. In the transcriptional autoregulation case, the output protein acts as a repressor that multimerizes and binds to its own promoter region to repress its own transcription (Figure 4A). In the post-transcriptional case, we model synthetic microRNA (miRNA) that binds to its cognate mRNA, simultaneously preventing translation and promoting degradation (Figure 5). In the remainder of this section we explore the effectiveness of these control mechanisms. The results provide specific guidelines for designing transcriptional and post-transcriptional autoregulatory networks for achieving best (or worst) suppression of protein variability.

#### Transcriptional autoregulation: High protein cooperativity achieves best suppression of variability

Simulations were made to study transcriptional autoregulation using the single gene regulatory network model in Figure 4A, in which the protein product represses its own transcription through a mechanism modeled by the Hill equation (which could represent protein oligomerization and DNA binding to block transcription); this is a common modeling framework in which binding is treated under a quasi-steady state assumption, and is valid when binding kinetics are substantially faster than the other reactions. We varied the Hill coefficient (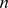) and the dissociation constant (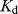) and observed the effect on the variability of the protein product. The quantity 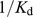 it is often associated with the feedback strength [35], [44]. Studies were made first with only intrinsic sources of variability and then with both intrinsic and extrinsic sources.

The intrinsic only case was studied with varying amounts of feedback strength and values of the Hill coefficient. We quantified variability as the relative squared coefficient of variation, 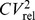, which is 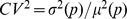 normalized by the 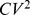 of the network without autoregulation (see Methods). According to this measure, whenever variability is less than 1 (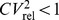), the negative feedback suppresses variability. Figure 4B shows the dynamics of protein numbers under the influence of intrinsic variability, which is represented by the means and standard deviations of the intrinsic noise distribution. For increasing feedback strength the means as well as the variances decreased. The measure of variability, 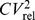, however, showed a minimum for intermediate feedback strength due to competition between different rates of decrease of the mean and the variance with increasing feedback strength. Reported are the variabilities calculated at the steady state; the data is plotted more concisely in Figure 4C (case 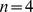).

We studied the effect of negative autoregulation on intrinsic protein variability under different Hill coefficients, 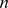, and different feedback strengths (Figure 4C). Several observations were made. Firstly, an optimal suppression of variability occurs for intermediate values of feedback strength. Secondly, there is a window of feedback strength for which autoregulation is successful in suppressing variability. And thirdly, the negative feedback only suppresses variability for Hill coefficients greater than one, while in the absence of protein cooperativity, the variability increases relative to that in a circuit without the feedback loop. These observations are consistent with those of Singh *et al.*, who did related analysis on a simplified model [35].

Next, we added extrinsic variability to the system by treating each parameter as a distribution across a population rather than as a constant value for all individuals. We studied the total protein variability resulting from a combination of intrinsic and extrinsic sources as a function of extrinsic variability. Initially, extrinsic variability was introduced in only the transcription rate (Figure 4D). Higher extrinsic variability led to greater suppression of expressed protein variability. This result is in agreement with Singh *et al.* [35]. As above in the intrinsic-only setting, the results suggest that high cooperativity in the protein is preferred to low cooperativity in order to achieve best suppression of variability when both intrinsic and extrinsic sources are acting on the system. Then we introduced variability in all other parameters individually (Figure S5). When the main source of extrinsic variability was in the gene copy number, translation rate, or in any of the degradation rates, we observed a similar result, namely that under higher extrinsic variability, self-repression suppressed protein variability better than a circuit without self-repression. The opposite was found for extrinsic variability in feedback strength or Hill coefficient: the higher the extrinsic variability, the less successful the loop was in suppressing the protein variability. In other words, this means that a noisy feedback mechanism is less successful in repressing protein variability than the feedback mechanism that is not noisy; this can be seen from a simple mathematical argument: a mixture distribution of individual components has higher variability than one of its individual components; the mixture corresponds to the total protein variability when extrinsic noise is present in the feedback strength or Hill coefficient, while its individual component corresponds to the intrinsic protein variability when no extrinsic noise is present in either of these parameters. Interestingly, the Hill coefficient is a parameter that is generally thought of as relatively unvarying for a given wild type protein.

In summary, transcriptional autoregulation is often thought of as a tool in synthetic biology to decrease, across a population of cells, the variability of the protein product of interest. Here we studied how to construct such an autoregulatory loop in order to achieve best suppression of variability. The results show that best suppression can be achieved when proteins are highly cooperative, and when extrinsic variability (either in transcription, translation, or degradation rates) across a population is large. Furthermore, there exists an optimal feedback strength that achieves best suppression.

#### Transcriptional regulation suppresses variability better than post-transcriptional regulation when extrinsic variability in translation and protein degradation rates is high

An important general problem in synthetic biology is to decide among candidate network topologies, and to select those with the best properties for implementation and potential tuning. Here we examine this question in the context of deciding between two topologies for suppression of variability of expressed protein. One topology incorporates transcriptional and the other post-transcriptional autoregulation. Crucially, we wanted our conclusions and design choices to be robust, in spite of our limited knowledge of extrinsic variability sources and network parameters.

Transcriptional autoregulation was modeled as above (Figure 4A), and post-transcriptional regulation by a model shown in Figure 5, in which a copy of mRNA and a synthetic miRNA are transcribed from a synthetically engineered DNA molecule [47]. After mRNA and miRNA have been spliced and processed, miRNA can bind to a complementary binding sequence on the mRNA molecule. Once bound, it can unbind or trigger miRNA-induced mRNA degradation, after which miRNA is recycled and can bind to other mRNA molecules [49], [50]. Binding and unbinding rates are denoted by 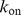 and 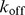, respectively, 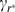 is the rate of miRNA-induced mRNA degradation, and 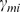 is the degradation rate of miRNA.

**Figure 6:**
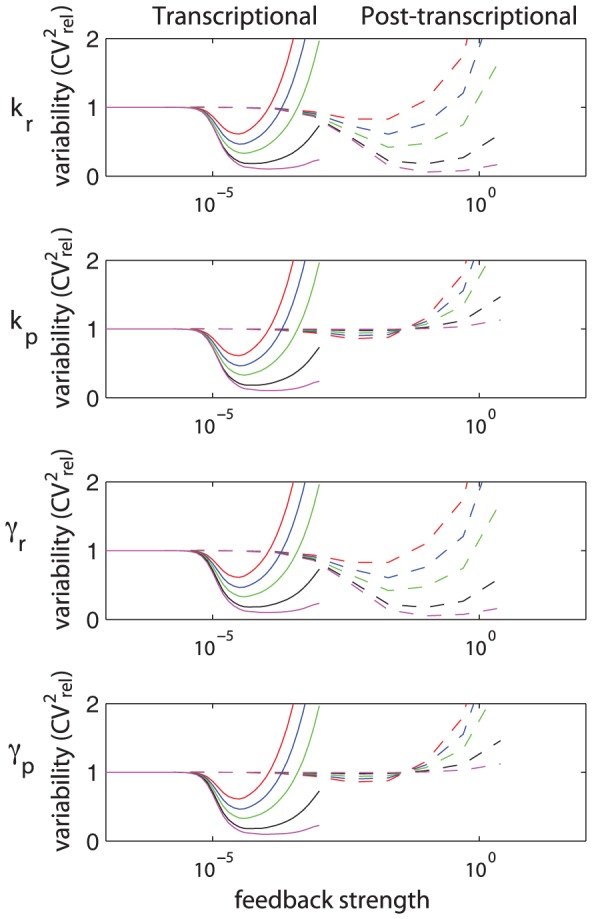
Comparison of total protein variability in transcriptional (solid line) and post-transcriptional (dashed line) self-repression circuits. Extrinsic variability in parameters (eqn. (13)): 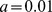 (red), 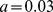 (blue), 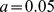 (green), 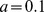 (black), and 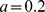 (magenta). Feedback strength in transcriptional self-repression model is 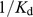, in post-transcriptional 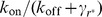.

To compare the effects of transcriptional and post-transcriptional self-repression on suppression of expressed protein variability, we introduced different amounts of extrinsic variability to different parameters and initial conditions of both models. Results showing suppression of total variability for different feedback strengths are shown in Figure 6. Each row shows how total variability in both models changes for different feedback strengths when extrinsic variability enters through each parameter individually. Increasing amounts of parameter variability in the transcriptional control model reduced protein variability for a window of feedback strength for all four parameters. By contrast, increasing amounts of parameter variability in the post-transcriptional control model reduced protein variability only for 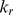 and 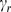 and increased it for 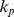 and 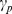. This latter observation is in line with our intuition; any variability in protein numbers that is due to extrinsic variability in 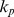 and 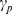 can not be suppressed by the post-transcriptional self-repression loop as translation and protein degradation in this case lie outside the self-repression loop. Moreover, effects for transcriptional control occurred at significantly smaller feedback strength than for post-transcriptional control, although note that these were computed differently for the two models. These results are summarized in Figure S5.

How can this knowledge help us choose between two designs of self-repression to suppress variability in the output of a synthetic circuit? Knowing that variability in translational parameters might be high [51], [52], our results suggest that the transcriptional self-regulation motif would be a “safer” choice to implement than post-transcriptional, if one aims to reduce the variability in the protein. However, if the sources and quantitative contributions of extrinsic variability were known and could be controled, one could make a more informed choice.

#### Post-transcriptional self-repression: Imperfect complementarity between miRNA and mRNA preferred to perfect complementarity

The mechanism of post-transcriptional regulation depends on the degree of complementarity between miRNA and mRNA [49], [53]; depending on complementarity, their interaction can either result in gene silencing through prevention of translation, or through induced mRNA degradation. If the complementarity is imperfect, binding and silencing will occur, but not mRNA degradation; perfect complementarity will lead to miRNA induced mRNA degradation. We model the effects of degree of complementarity in our post-transcriptional model (Figure 5) through the parameter 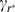. In this section we studied what values of 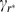 lead — and therefore what degree of complementarity leads — to best suppression of variability.

**Figure 7:**
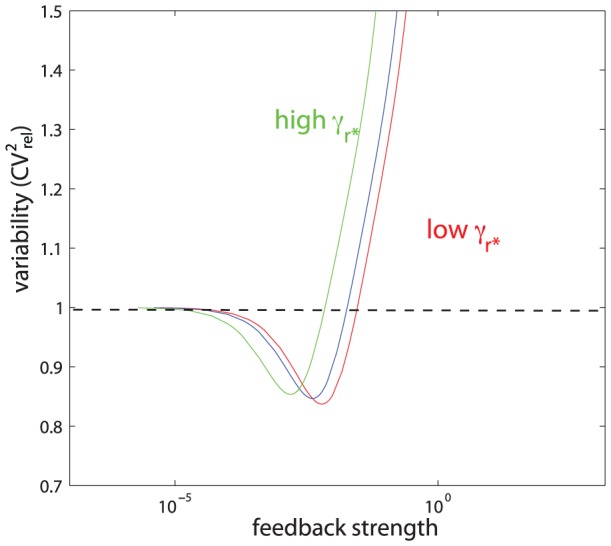
Variability in post-transcriptional circuit for different values of 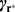
 **.** Parameter 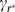 depends on the level of complementarity between mRNA and miRNA. 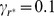 (red), 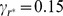 (blue), and 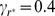 (green). Feedback strength 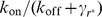. Intrinsic variability only was used.

The results are shown in Figure 7. The measure of intrinsic protein variability is plotted against different values of feedback strength. The results show that for low rates of miRNA-induced mRNA degradation, 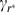, there is wider and stronger window of reduced intrinsic stochasticity than for high rates of 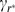. The same was observed also when high extrinsic variability was present in the system, although the differences were less pronounced (Figure S6). These results suggest that best noise suppression is achieved if miRNAs repress translation but do not promote mRNA degradation, and thus that imperfect complementarity between mRNA and miRNA is preferred to perfect complementarity.

#### miRNA and mRNA transcribed on a single transcript are best for suppression of protein variability

**Figure 8:**
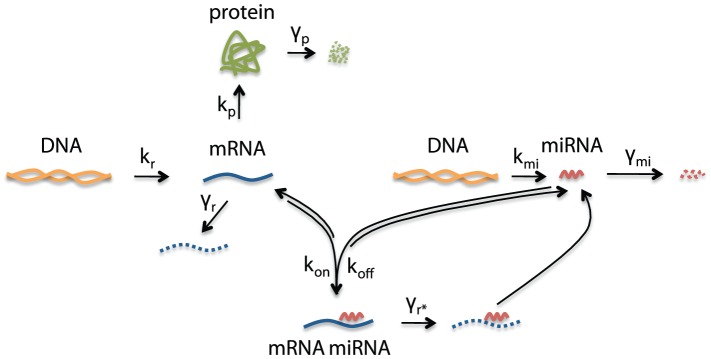
Post-transcriptional repression model with miRNA transcribed on a different transcript than mRNA.

**Figure 9:**
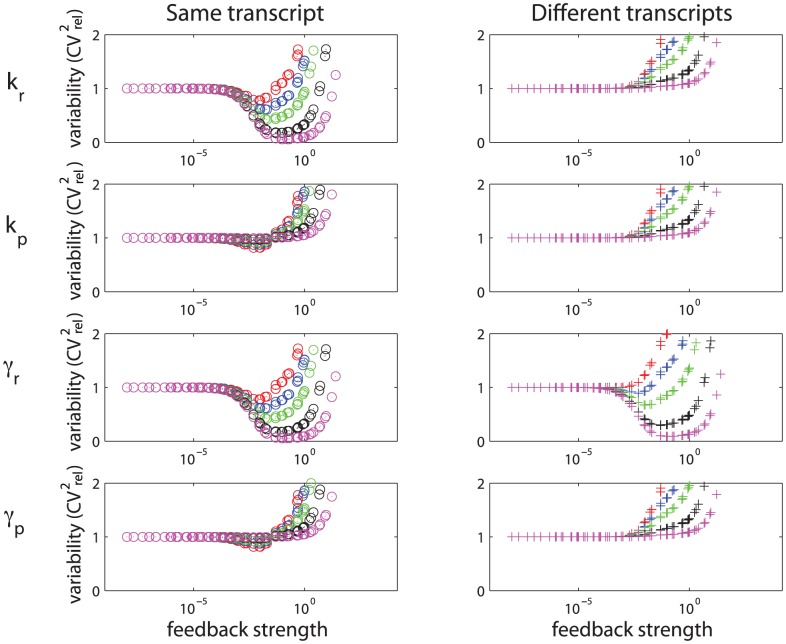
Comparison of total protein variability in circuits with mRNA and miRNA on the same (o) or different (+) transcripts. Extrinsic variability in parameters (eqn. (13)): 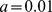 (red), 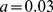 (blue), 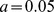 (green), 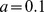 (black), and 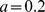 (magenta). Feedback strength is 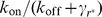.

In the post-transcriptional model so far, mRNA and miRNA were transcribed on the same transcript. We next compared the ability of post-transcriptional repression to suppress variability when mRNA and miRNA were transcribed together, to when they were transcribed on two separate transcripts (Figure 8). We chose the values of transcription rates of mRNA and miRNA to be equal in both models. The crucial difference between both circuits is that mRNA and miRNA molecules are produced in a correlated manner when encoded on the same transcript, and in an uncorrelated manner when encoded on different transcripts. Figure 9 shows that for extrinsic variability in parameters 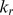, 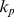, and 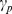, lower variability was obtained for the “same-transcript design” compared to the “different-transcripts design”, with results for 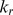 especially dramatic. However, both designs were equally successful in reducing protein variability under extrinsic variability in parameter 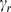. This suggests that the latter circuit was less successful in reducing variability than the former for most extrinsic sources; this observation is in agreement with Osella *et al.* [46]. Furthermore, for the same-transcript design, variability in 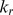 and 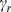 are especially effective in suppressing protein variability.

#### Low cooperativity in miRNA achieves most robust suppression of variability

**Figure 10:**
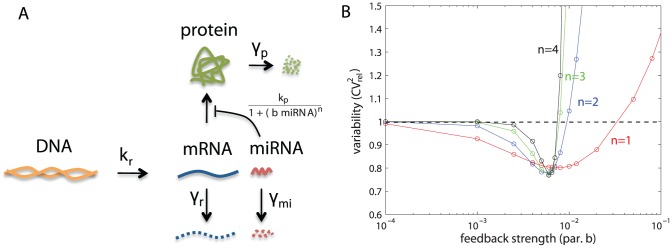
Results for post-transcriptional self-repression modeled by a Hill function. (A) Single gene expression model with post-transcriptional self-repression modeled by a Hill function. (B) Effect of self-repression on variability with only intrinsic sources of variability, for Hill coefficient values of 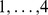.

It is currently unknown whether cooperativity between miRNA molecules exists, what its mechanism would be, or how one could go about designing it. Nevertheless, it has been suggested that cooperative models might be more appropriate for modeling regulation by miRNA than non-cooperative models [54]. These models use a Hill function, which circumvents the need for modeling the precise molecular mechanism. Here we used one such model (Figure 10A) to study the effect of miRNA cooperativity on suppression of variability. High cooperativity was observed to reduce variability slightly more in magnitude; however, and more significantly, low cooperativity was successful in reducing variability for a much broader range of feedback strengths (Figure 10B). In other words, the suppression of noise in the absence of miRNA cooperativity was more robust to varying feedback strength. Therefore, depending on how difficult it is to design (or know) a precise feedback strength, low cooperativity might be preferred to high cooperativity.

#### Correlated extrinsic variability

Little knowledge exists for biological systems about the sources and amounts of extrinsic variability, which makes it uncertain how one should specify the covariance matrix 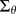. To this point of the current report, we have considered the covariance matrix to be diagonal, corresponding to independent parameter variation. However, some biological parameter variability might be correlated; for example, if differences in cell size drive protein variability through changes in concentration of the transcriptional, translational, and degradation machinery, then transcriptional, translational, and degradation rate-constant variability could be correlated. The framework introduced here allows arbitrary parameter correlations originating from extrinsic variability. While this may seem like “extra” parameters required by this framework, it is perhaps more accurate to say that they are required to properly describe the underlying biophysics, and the method we present exposes them explicitly.

To explore how correlations in parameter variability affect overall gene expression variability, we next carried out model studies with different covariance matrices and analysed the results. We are interested in the question of how correlations in extrinsic variability affect the ability of autoregulatory circuits to suppress total variability in protein concentration. Are rate correlations important to understand and measure in order to understand total biological variability, or are they minor contributors? Are there particularly favorable correlation properties of kinetic rates across a population of cells that suppress (or enhance) variability? How robust is noise suppression in transcriptional relative to post-transcriptional self-repression circuits to different parameter distributions across cells?

**Figure 11:**
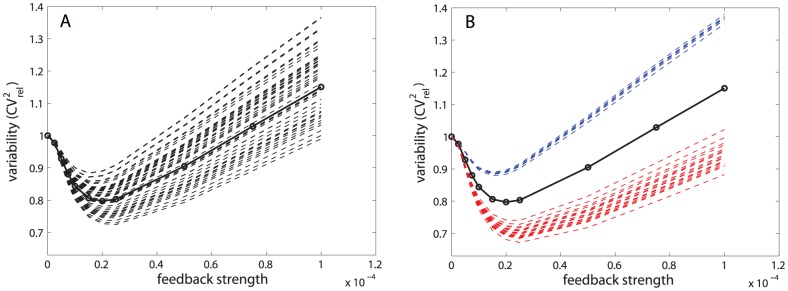
Total variability resulting from non-diagonal covariance matrices. (A) Simulations of total variability resulting from 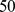 representative random correlation matrices selected from 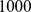 runs for visual clarity (dashed line) and a diagonal correlation matrix (solid line). (B) From a total of 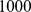 random correlation matrices, those resulting from correlation matrix (eqn. 6) are shown in red, and those correlated oppositely in blue. Simulation resulting from a diagonal correlation matrix is represented by a solid black line.

To answer these questions, we first generated covariance matrices with fixed diagonal elements but random off-diagonal elements. This corresponds to the same total variation for each parameter but different covariation among the parameters for each trial. We simulated the total variability in protein numbers in both transcriptional and post-transcriptional self-repression circuits using 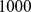 random matrices each. The variability of a system with independent extrinsic parameter variability (all zeros off the diagonal) is taken as a reference and is plotted as the solid line in Figure 11A. When we included correlated extrinsic variability, the suppression was better or worse (indicated by dashed lines appearing on both sides of the solid line). To address the question of what type of extrinsic variability allows for better or worse suppression, we compared the total variability resulting from correlated parameters to that from uncorrelated parameters for each pair of parameters (Figure S7). This allowed us to hypothesize that the following correlation matrix would result in strong suppression of variability in a transcriptional autoregulatory network (note the order of indices is 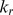, 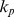, 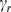, 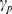 for rows and columns),
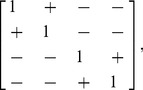
(6)where a 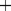 sign means the parameter pair is correlated and 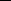 sign that it is anti-correlated; likewise, the opposite correlation matrix was hypothesized to result in weak suppression (and even enhancement) of variability. This hypothesis was validated for randomly generated variances (i.e., diagonals of the covariance matrix). For every randomly generated variance vector, covariance matrices with correlation properties defined by (6) with symmetric off-diagonal elements choisen randomly between zero and one resulted in better suppression (red) than independent extrinsic variability (black), and correlation properties opposite to (6) resulted in worse suppression (blue; Figure 11B). This result suggests that transcriptional autoregulatory networks are best in suppressing variability in systems where (i) transcription and translation rates are correlated, (ii) mRNA degradation and protein degradation rates are correlated, and (iii) all other pairs of kinetic rates are anti-correlated.

Using the same approach we found that the post-transcriptional autoregulation circuit strongly suppressed variability if their extrinsic noise environment was determined by the following correlation matrix pattern
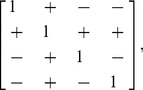
(7)and weakly suppressed (and even enhanced) variability for the opposite correlation matrix pattern (Figure S8). Therefore, extrinsic variability with (i) positively correlated transcription and translation rates, (ii) positively correlated translation and mRNA degradation, (iii) positively correlated translation and protein degradation rates, and (iv) all other pairs anti-correlated, would allow for best suppression of variability in the post-transcriptional autoregulation circuit. This tells us that different circuits suppress variability better or worse depending on the intra-cellular environments that determine correlations between kinetic parameters.

**Figure 12:**
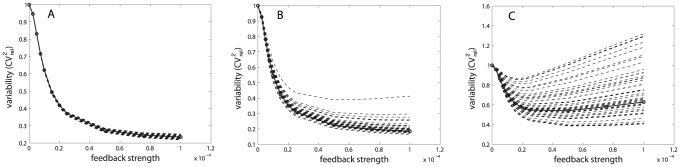
Small, medium and large differences in total protein variability resulting from different covariance matrices. Simulations of total variability resulting from 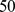 different random correlation matrices selected from 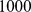 runs for visual clarity (dashed line) and a diagonal correlation matrix (solid line). The total variability shows different degrees of sensitivity to correlation matrices, depending on parameter variances: (A) 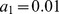, 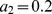, 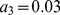, 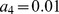 (B) 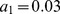, 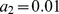, 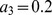, 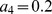 (C) 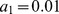, 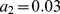, 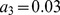, 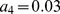.

We next investigated the question whether particular patterns of parameter variability could control the effect of parameter covariation on overall protein variability. We were curious whether some diagonals could enhance or suppress the effect of off-diagonals in creating protein number variability. To address this question we sorted diagonals according to the similarity of the outputs they produced for different covariance matrices, and looked at which diagonal combinations resulted in small, medium, and large differences in total protein variability as the correlation pattern was varied (Figures 12A, B, C, respectively). For the transcriptional self-repression circuit, we observed that when extrinsic variability was dominated by variability in transcription or translation, with variability in all other parameters small, then different covariance matrices gave very similar total variability results (Figure 12A is one example; other examples are given in Text S1). In contrast, when extrinsic variability was a combination of small and intermediate sizes for different parameters, with none of them clearly dominating, then different covariance matrices gave very different total variability results. These results imply that, depending on the amount of extrinsic variability, it is either important to understand correlations between parameters (as they effect the ability to suppress variability, Figure 12C) or not important (as variability will be suppressed to the same extent regardless of the correlations between parameters, Figure 12A). For design purposes, where it may be difficult to even know and certainly to control covariation fully, it may be useful to explicitly choose combinations of variation that minimize the effect of covariation.

The ability to specify arbitrary parameter correlations in extrinsic variability is a valuable property of this modeling framework. This property, combined with an efficient simulation framework, allows exploration of how total variability of the model output depends on different sources and properties of extrinsic variability. Questions that remain to be answered include what is the biological explanation for this phenomena, and how important a role it plays in the detailed workings of biological systems.

#### Transient variability behaves differently than steady-state variability

Up to this point in the current report, we have studied distributions of variability in the steady state; however, the framework allows the same analysis for any time point in the simulation. Our observations are consistent with those of Tao *et al.* [55], who showed theoretically that intrinsic stochasticity in transient states might behave differently than intrinsic stochasticity in the steady state. In addition, our calculations show that this result also holds for the combination of intrinsic and extrinsic variability. For example, while we saw earlier that transcriptional self-repression with Hill coefficient 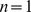 could not suppress variability in the steady state, the variability could be suppressed in transient states (Figure S9). Similarly, for higher Hill coefficients, especially under strong feedback, variability might be suppressed in the transient but not in the steady state (Figure S10). This phenomenon has recently been observed experimentally in the NF-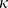
B signaling pathway [56]. Interestingly, for both transcriptional and post-transcriptional self-repression, the qualitative effect of different levels of extrinsic variability on the system (results shown for transcriptional case in Figure S11) was the same in the steady and transient states (steady state in Figure S5). Taken together, these observations strongly caution that conclusions about steady-state variability properties cannot straightforwardly be extrapolated to transient states.

## Discussion

Variability is ubiquitous to biological systems, and yet it is not well understood. In order to engineer and intervene in such systems successfully, variability must be taken into account in planning and interpreting experiments and in designing synthetic networks. Here we have introduced a framework for modeling and simulation of dynamical systems under both intrinsic and extrinsic sources of variability. One reason that understanding and treating variability is so especially important for synthetic biology is that one seeks to design circuits and devices that can perform successfully across a population of cells and variety of experimental conditions; without understanding variability and fluctuations, one risks building devices or designing therapies that only perform well in a fraction of cells (or patients) or only under limited environmental conditions.

Here we addressed the problem of constructing circuits that exhibit low variability across cells. We investigated transcriptional and post-transcriptional designs of self repression, and studied their relative promise for noise suppression. We explored their behavior for a range of feedback strengths and other design parameters, and for various hypothetical amounts and types of extrinsic variability. We derived a set of design principles for best noise suppression in a protein concentration of interest. We showed that transcriptional autoregulation is more successful than post-transcriptional in suppressing noise under a wide range of intrinsic and extrinsic variability levels and conditions. The following design principles were shown to best suppress protein concentration variability: (i) high cooperativity in protein binding to DNA and low cooperativity in miRNA binding to mRNA, (ii) imperfect complementarity between miRNA and mRNA was preferred to perfect complementarity, and (iii) correlated expression of mRNA and miRNA — for example, on the same transcript. We further showed that correlations in kinetic parameters between cells affected the ability to suppress variability, and that variability in transient states did not necessarily follow the same principles as variability in the steady state. Also, we note that biochemical design can not avoid fundamental limits on the lower bound of stochastic fluctuations [57]. In this work we have focused on one objective, which is to decrease variability of protein expression. In synthetic biology applications, one might be interested in additional objectives, such as maximizing the mean protein expression, or keeping the mean fixed while suppressing variability etc. The same analysis can be performed for such objectives, and could potentially lead to different guidelines.

Our novel modeling and simulation framework combines intrinsic and extrinsic sources of variability by combining the 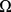-expansion with the unscented transform. In contrast to few previous methods, our framework is based on a deterministic simulation with ordinary differential equations. One advantage of our method is that it incorporates extrinsic variability by repeated simulation of the same intrinsic model for carefully selected parameter combinations where the number of simulations increases linearly with the number of extrinsic factors (see Methods), rather than generating a new model and exponentially increasing the model size for each new extrinsic factor [33]. This not only increases computational efficiency, but makes possible analysis with a wide variety of available tools. It also allows for screening across a large range of parameters (such as feedback strength, 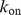 and 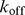 rates, and unknown sources and quantities of extrinsic variability). The new approach is well suited to support the design of synthetic circuits from building blocks with partially unknown kinetic properties but that will perform desired functions robustly in variable intracellular and extracellular environments and across a population of cells. An important aspect of the framework is that it can be applied to study variability and dynamics at transient as well as steady states; we have shown that variability might behave differently in both regimes, which reiterates the importance of developing and using tools that are applicable beyond steady-state regimes.

The computational efficiency of the framework, however, comes at a cost of approximation; macroscopic dynamics remain non-linear, but intrinsic and extrinsic variability distributions are both approximated by the first two moments. We have shown that transient and steady-state distributions resulting from our approximation framework closely fit to exact distributions simulated by a Gillespie algorithm and parameter sampling. We note once more that the 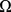-expansion is derived under the assumption of large numbers of molecules and we recommend the results to be tested by more accurate methods, especially for low molecule numbers. For example, a sufficient number of Gillespie runs provides the exact distribution [14]; another powerful technique, the Langevin approximation, can be used to obtain more accurate than 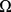-expansion [20], although still approximate estimates of the variance at a steady state, and its disadvantage is that it requires monitoring of the convergence. An important point, however, is that we have focused less on calculating exact quantitative values of variability, and more on what changes in the network drive variability increases or decreases. Our approximation framework is sufficient for addressing this question, and due to computational efficiency in both steady and transient states it is even advantageous compared to exact simulation. We have applied the framework to systems with a single steady state, but there is scope for extending the framework to oscillatory and multimodal systems [25]–[27]. Derivation of improved versions of the 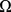-expansion is an area of active research, for example accounting for slow and fast variables [58] or increasing accuracy for small number of molecules [59]. By developing better approximation frameworks for intrinsic noise modeling, our combined intrinsic-extrinsic method will increase in accuracy, too. Furthermore, while the dynamics originating from intrinsic variability was modelled by the 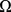-expansion approximation framework in this manuscript, a similar framework could be constructed based on other approximation modeling approaches for intrinsic variability, for example the moment closure method [60] or mass fluctuation kinetics [16]. The extrinsic variability modeled through parameter distributions in this manuscript was assumed to be constant in time (i.e., static), and a natural extension would be to introduce time-varying parameter distributions (i.e., dynamic) [31], [61]. Because of this, for example, our method in its current form can not be used to quantify the dependence of the total output variation on the lifetime of extrinsic fluctuations [32].

There are different sources of biological variability and uncertainty; a careful interpretation and appropriate inclusion in a mathematical model is crucial to elucidate the dynamics of biological variability and understanding of these different levels of biological complexity. Firstly, there are intrinsic and extrinsic sources of variability (discussed at length above). Secondly, there is parameter uncertainty, which encompasses our limited knowledge of kinetic rate values. This uncertainty is commonly included in biological models and simulations as a distribution of kinetic values, however, it is important to treat it separately from extrinsic variability, which is also modeled by treating parameters as distributions. An example of appropriate mathematical treatment is a hierarchical Bayesian model [33], [62]. A further type of uncertainty is model uncertainty, by which we refer to our uncertainty in model topology; the present manuscript does not address this type of uncertainty. And thirdly, when modeling or analyzing experimental biological data, one also needs to account for measurement noise (or measurement error). In order to infer sources of variability and reduce uncertainty in parameters, there is a need for inference techniques for models that account for these different levels of biological variability, uncertainty, and measurement noise.

The presented modeling framework is only the first step in the systems biology cycle of modeling, designing experiments, and updating the model by inference from collected data. Based on this modeling framework, we are now developing tools that account for variability also in the experimental design stage, and for updating the models from noisy data through parameter inference and model selection algorithms. These techniques will allow us to better explore and account for biological variability in synthetic biology, a ubiquitous aspect of biological systems that is in practice often neglected.

## Methods

### The 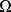-expansion

The 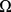-expansion is an approximation of the master equation. It separates the macroscopic part of dynamics from the fluctuations around it, describing each of these parts by a set of ODEs [13]. The resulting 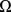-expansion model approximates the first two moments of the intrinsic noise distribution in the form of ODEs for each mean, variance, and covariance; for 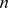 species in the model, the 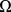-expansion generates 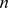 equations for the means, 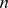 equations for variances, and 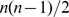 equations for covariances between all pairs of species.

Readers interested in a theoretical derivation of the 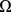-expansion modeling framework are referred to van Kampen [13]. As a brief summary we note that the 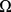-expansion is derived assuming that the macroscopic part of dynamics can be separated from the fluctuations centred around it, and that the fluctuations scale as the square root of the number of molecules [63]. This is captured by the following ansatz: 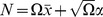, where 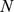 is the number of molecules, 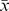 is the macroscopic concentration and 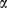 the fluctuations. Parameter 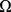 represents the system size and can be thought of as proportional to the volume. This ansatz is introduced into the master equation governing the evolution of probability distribution 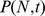 and then the master equation is Taylor-expanded around 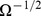. The terms of order 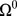 collected together determine equations governing macroscopic dynamics, and the terms of order 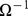 the equations for dynamics of fluctuations.

The resulting set of ordinary differential equations describing macroscopic (or average) behavior is
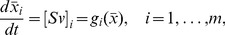
(8)where 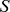 is the stoichiometry matrix and 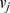 are the reaction propensities of reactions 
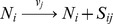
. The ODE for the covariance matrix of the fluctuations, 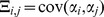, centred around 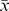 is
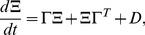
(9)where 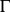 is a Jacobian of a deterministic system given by equation (8), evaluated along the macroscopic trajectory, 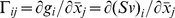 and is called the *drift*. Matrix 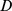 is the *diffusion matrix*, 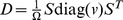. Equations (8) and (9) together form the 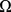-expansion model, which approximates the first two moments (mean 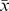 and covariance matrix 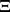) of the distribution that solves the master equation. The 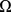-expansion model is a deterministic and approximate description of intrinsic noise distribution dynamics in time.

### Visualizing multivariate normal distributions as ellipses

We visualised the multivariate normal distributions 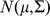 as ellipses. The ellipse is one of the contours that represent equal probability mass of the distribution. In general, these ellipses are constructed from eigenvalues 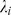 and eigenvectors 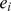 of the covariance matrix 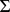. They are centred at mean 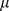, their axis directions are determined by the eigenvectors and axis sizes by the eigenvalues, 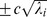. Different values of 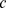 specify different contours. In our figures we choose 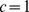 unless otherwise specified. In particular, the ellipse determined by equation
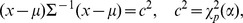
represents the multivariate analogue of the “confidence interval” containing 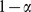 of the probability mass of the distribution. For example, a bivariate (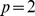) normal distribution represented by an ellipse with axes lengths 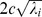, 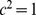, contains 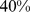 of distribution, and an ellipse containing 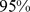 of a distribution has axes of lengths 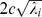, 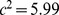.

### The Unscented Transform

**Figure 13:**
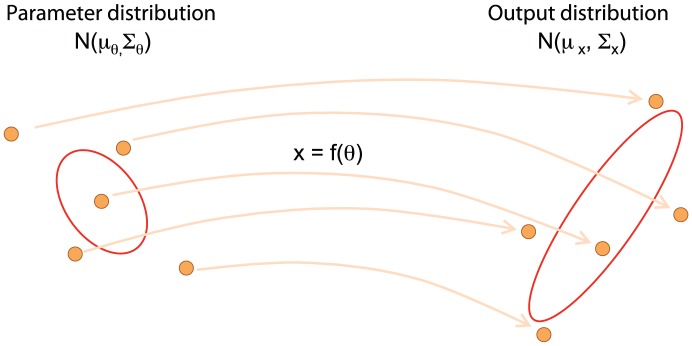
Schematic representation of the unscented transform. Adapted from ref. [36]. The UT provides a mechanism for approximating the output distribution 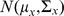 obtained by propagating the input parameter distribution 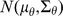 through the function 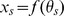. It does this by deterministically choosing a minimal set of sample points (called sigma points; filled circles to left) from the parameter distribution, 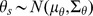. Sigma points are then propagated through function 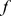, 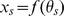 (filled circles to right), and re-assembled into a Gaussian distribution on the output, 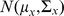, which approximates the output distribution by the first two moments (ellipse to right).

The unscented transform (UT) is an algorithm that efficiently propagates normally distributed inputs through a nonlinear function to obtain a distribution on the function outputs. Figure 13 provides an overview of the UT.

Here 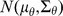 was the multivariate normal distribution on the space of parameters and initial conditions 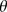 of dimension 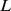. We deterministically sampled from 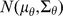 a set of *sigma points*

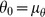
(10a)

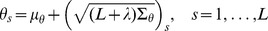
(10b)

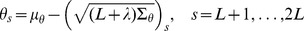
(10c)and assigned weights
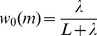
(11a)

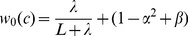
(11b)

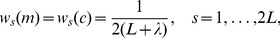
(11c)where 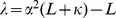. See ref. [36] for guidelines on how to choose parameters 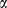, 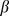 and 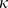; we chose 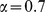, 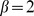, 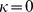. The above weights define the *scaled* unscented transform, which is the generalized version of the unscented transform algorithm [64].

Function 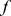 was evaluated for every sigma point,
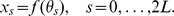



The model outputs 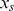 obtained were then reconstructed into a multivariate normal distribution with mean 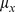 and covariance matrix 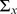 determined by the following equations:
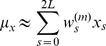
(12a)

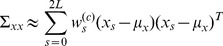
(12b)

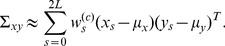
(12c)


We note that the UT is computationally much more for efficient compared to random sampling; UT only requires 2L+1 simulations (one for each sigma point), while this number needs to be considerably higher for exhaustive sampling, especially for high dimensional parameter spaces. In terms of accuracy, the UT estimates the first two moments accurately to second order in the Taylor series expansion for any nonlinear function [65], [66]. However, should one sample 2L+1 points randomly and propagate them, one will not achieve this accuracy and therefore exhaustive sampling is needed to achieve the same level of accuracy as the UT (the exact number of simulations needed depends on the specific system and space dimension of parameters and initial conditions).

The UT can also be used to propagate log-normal distributions (see Text S1 for details), and mixtures of (log-)normal distributions.

### A measure of variability

The variance of protein numbers is not by itself a good measure of variability, as it does not account for the protein mean; for example, with increasing feedback strength in an autoregulatory network, the fluctuations in protein numbers decrease, but the mean number decreases, too. If absolute numbers of proteins are small, then protein variance becomes relatively large compared to its mean. For most applications, both mean and variance of the protein numbers should be included in the measure of variability. Here we use the squared coefficient of variation, 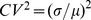 [4], normalised by 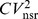, the measure of variability without self-repression (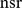 stands for no self-repression), to obtain the relative coefficient of variation, 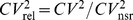. Therefore, 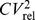 will have value 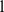 when no self-repression is present in the model. Whenever variability according to this measure is less than 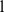, self-repression is effective in suppressing variability.

### Specifying extrinsic variability through parameter distribution

Extrinsic variability is described by the covariance matrix 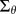 of parameters and initial conditions. Extrinsic variability can enter the system though variability in a single parameter or a combination of parameters, and parameters can be arbitrarily (anti-)correlated. A diagonal covariance matrix assumes independence between all pairs of parameters. (Anti-)correlations between parameters are modeled by including non-zero off-diagonal terms. The covariance matrix is symmetric by definition.

We defined the amount of extrinsic variability by specifying a coefficient of variation for each parameter,
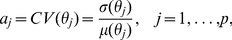
(13)and set the diagonal entries of the matrix 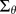 to 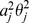. The parameter dimension is denoted by 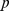. We chose different amounts of extrinsic variability 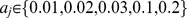, as noted in specific figure captions.

When independence between parameters was assumed, the off-diagonal terms were set to zero. Additionally, we also created arbitrarily correlated parameter distributions by generating random covariance matrices in the following manner. We first generated a random correlation matrix of size 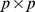, by generating a symmetric matrix with diagonal elements 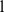 and off-diagonal elements sampled from a uniform distribution 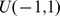, and then finding the nearest valid correlation matrix 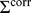 by using Higham's algorithm [67]. This correlation matrix was transformed to a covariance matrix by multiplying each element of the correlation matric by the standard deviations corresponding to its row and column, 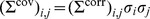, where 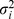, were the variances (i.e., diagonal elements of the covariance matrix).

### Parameter values and units

The units are in the form of number of molecules (or number of molecules/sec) rather than concentration (or concentration/sec). This allows us to more easily to compare the exact Gillespie simulation with the output of our approximation framework.

Parameters 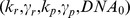 used in examples in figures were set to 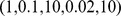 and 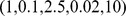. Parameters that determine the feedback strength, 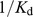 and 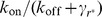, were varied as indicated in the figures. The Hill coefficient 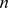 took integer values between 1 and 4. The units of zeroth-order parameters were *number of molecules per second* (or *molecules per second*), first-order parameters *per second* and second-order parameters *per molecule per second*. The conclusions were checked to hold for other parameter combinations, in particular 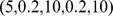, 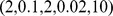, 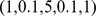, 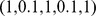, and 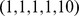.

## Supporting Information

Figure S1Combined framework using the 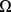-expansion and the UT allows for efficient calculation of total variability.(EPS)

Figure S2Comparison of distributions generated by 10,000 stochastic Gillespie runs (blue) with the 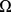-expansion simulation (green) at steady state 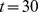 and a transient time point 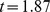. (A) Projected univariate mRNA and protein distributions. (B) Bivariate distribution between mRNA and protein. The inner green ellipse principal axis sizes correspond to sizes of eigenvalues of the covariance matrix (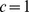 and it contains 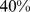 of the probability mass; see Methods). In the outer green ellipse, axis sizes have been scaled so that the ellipse represents the 95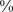-confidence interval contour, i.e., the contour containing 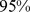 of the probability mass of the corresponding multivariate normal distribution.(EPS)

Figure S3Comparison of distributions generated by 1,000,000 stochastic Gillespie runs with varying transcription rate 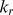 with coefficient of variation 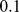, i.e. 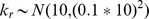 (black) with the combined 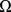-expansion and UT simulation (red) at steady state 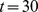 (top row) and a transient time point 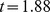 (bottom row).(EPS)

Figure S4Comparison of distributions generated by 1,000,000 stochastic Gillespie runs with varying parameters 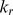, 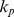, 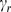, and 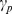 independently with coefficient of variation 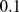 (black) with the combined 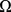-expansion and UT simulation (red) at steady state 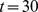 (top row) and a transient time point 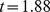 (bottom row).(EPS)

Figure S5The effects of extrinsic variability in individual parameters on the total protein variability. An arrow upwards (downwards) means that with increasing amount of extrinsic variability in the respective parameter, the total variability in the protein (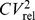) increases (decreases).(EPS)

Figure S6Comparing total protein variability in post-transcriptional circuit for different values of 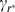, the parameter that depends on the level of complementarity between mRNA and miRNA. 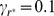 (solid line) 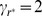 (dashed line). Extrinsic variability in parameters: a = 0.01 (red), a = 0.03 (blue), a = 0.05 (green), a = 0.1 (black), and a = 0.2 (magenta). Feedback strength is 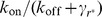.(EPS)

Figure S7Total variability of transcriptional self-repression resulting from different correlations of extrinsic variability. Lines depicting variability resulting from positively correlated (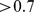) extrinsic variability in a specified parameter pair are colored in red, and lines resulting from anti-correlated (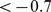) extrinsic variability between the parameter pair are colored in blue. Parameters determining the diagonal values of the covariance matrix used in this example were 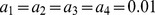. We note that for a few other sets of diagonal values, the distinction between blue and red for some parameter pairs was less clear than for this example.(EPS)

Figure S8Total variability of post-transcriptional self-repression resulting from different correlations of extrinsic variability. Lines depicting variability resulting from positively correlated (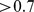) extrinsic variability in a specified parameter pair are colored in red, and lines resulting from anti-correlated (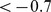) extrinsic variability between the parameter pair are colored in blue. Parameters determining the diagonal values of the covariance matrix used in this example were 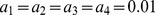. We note that for a few other sets of diagonal values, the distinction between blue and red for some parameter pairs was less clear than for this example.(EPS)

Figure S9Intrinsic transient variability can be suppressed even when intrinsic steady-state variability cannot be suppressed. Shown is the total variability of the protein in the transcriptional autoregulatory network with Hill coefficient 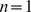 for different feedback strengths. At steady state (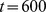) the autoregulatory motif cannot suppress variability, which we see from the fact that 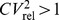. However, in transient states (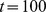 and 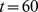) the total variability is suppressed compared to that in a network without self-regulation. This follows from the fact that 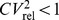 for certain feedback strengths.(EPS)

Figure S10Transient variability can be suppressed better than steady-state variability. Across all feedback strengths the minimum variability reached in transient states (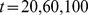) is lower than the minimum variability reached in the steady state (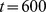). For high feedback strengths (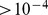) the steady-state variability is greater than transient variability.(EPS)

Figure S11In transient states the qualitative effects of extrinsic variability on total variability agree with those in steady states (Figure 5). Results shown for 4 parameters in a transcriptional autoregulation. Extrinsic variability in parameters: a = 0.01 (red), a = 0.03 (blue), a = 0.05 (green), a = 0.1 (black), and a = 0.2 (magenta). Feedback strength is 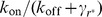.(EPS)

Text S1 Contains a more detailed description of the procedure for calculating total variability from the combined model with intrinsic and extrinsic sources of variability, examples of extrinsic variability that produce small and large differences in total variability, and notes on using the unscented transform to propagate log-normal distributions through a nonlinear function.
(PDF)
