# Supplementary material for: Combined Model of Intrinsic and Extrinsic Variability for Computational Network Design with Application to Synthetic Biology
Source: PLoS Comput Biol. 2013 Mar 28;9(3):e1002960. doi: 10.1371/journal.pcbi.1002960 (PMC3610654; doi:10.1371/journal.pcbi.1002960)
Supplement: Text S1 — Contains a more detailed description of the procedure for calculating total variability from the combined model with intrinsic and extrinsic sources of variability, examples of extrinsic variability that produce small and large differences in total variability, and notes on using the unscented transform to propagate log-normal distributions through a nonlinear function. (PDF) [file pcbi.1002960.s012.pdf]

Text S1: Combined model of intrinsic and extrinsic variability for computational network design with application to synthetic biology

Tina Toni, Bruce Tidor

Calculation of total variability from the output of the combined model

Here we provide further mathematical details on the calculation of total variability. Intrinsic variability is determined by the Ω-expansion model, and extrinsic variability by the distributions of parameters and initial conditions, which are efficiently propagated through the model by using the UT. We assume parameters and initial conditions are normally distributed, $\theta \sim N(\mu_\theta, \Sigma_\theta)$, and apply the UT to propagate the parameter distribution through the Ω-expansion model. This requires simulations of the Ω-expansion model at $2L + 1$ parameter realizations (i.e., sigma points) — where L is the dimension of parameters θ — which are then "superimposed" (by using the weights determined by the UT algorithm) to construct the output of the model.

The combined framework is schematically depicted in Figure S1. The output of the model is a Gaussian distribution in the space of model output at time t,

$$P\left(\left[\begin{array}{c}\bar{x}\\ \Xi\end{array}\right]\right) = N\left(\left[\begin{array}{c}\mu_{\bar{x}}\\ \mu_{\Xi}\end{array}\right], \left[\begin{array}{cc}\Sigma_{\bar{x},\bar{x}} & \Sigma_{\bar{x},\Xi}\\ \Sigma_{\Xi,\bar{x}} & \Sigma_{\Xi,\Xi}\end{array}\right]\right). \quad (1)$$

where $\bar{x}$ denotes species means, and Ξ denotes variances and covariances. $\mu_{\bar{x}}$ is the mean value of species, μ_Ξ is the mean of variances and covariances of all intrinsic components, and $\Sigma_{\bar{x},\bar{x}}$ is the variance (due to parameter distributions or extrinsic sources of noise) of species means. The total mean of the species equals $\mu_{\bar{x}}$ (which follows from eq. (4)), and the total variability (following eq. (5)) equals

$$Var^{tot} = \mu_\Xi + \Sigma_{\bar{x},\bar{x}}. \quad (2)$$

These equation allow us to calculate the total variability very efficiently, directly from model (1), which is obtained by only $2L + 1$ simulations of the deterministic Ω-expansion model.

For the single gene expression model, the distribution over $(\bar{m}, \bar{p}, Var_m, Var_p, Cov_{m,p})$ at each time t is given by the combined model (1)

$$P\left(\left[\begin{array}{l}\bar{m}\\ \bar{p}\\ Var_m\\ Var_p\\ Cov_{m,p}\end{array}\right]\right) =$$

$$N\left(\begin{bmatrix} \mu_{\bar{m}} \\ \mu_{\bar{p}} \\ \mu_{Var_m} \\ \mu_{Var_p} \\ \mu_{Cov_{m,p}} \end{bmatrix}, \begin{bmatrix} \Sigma_{\bar{m},\bar{m}} & \Sigma_{\bar{m},\bar{p}} & \Sigma_{\bar{m},Var_m} & \Sigma_{\bar{m},Var_p} & \Sigma_{\bar{m},Cov_{m,p}} \\ \Sigma_{\bar{p},\bar{m}} & \Sigma_{\bar{p},\bar{p}} & \Sigma_{\bar{p},Var_m} & \Sigma_{\bar{p},Var_p} & \Sigma_{\bar{m},Cov_{m,p}} \\ \Sigma_{Var_m,\bar{m}} & \Sigma_{Var_m,\bar{m}} & \Sigma_{Var_m,Var_m} & \Sigma_{Var_m,Var_p} & \Sigma_{Var_m,Cov_{m,p}} \\ \Sigma_{Var_p,\bar{m}} & \Sigma_{Var_p,\bar{m}} & \Sigma_{Var_p,Var_m} & \Sigma_{Var_p,Var_p} & \Sigma_{Var_p,Cov_{m,p}} \\ \Sigma_{Cov_{m,p},\bar{m}} & \Sigma_{Cov_{m,p},\bar{m}} & \Sigma_{Cov_{m,p},Var_m} & \Sigma_{Cov_{m,p},Var_p} & \Sigma_{Cov_{m,p},Cov_{m,p}} \end{bmatrix}\right),$$

and the total variability is given by the variance-covariance matrix

$$\begin{bmatrix} Var_m^{tot} & Cov_{m,p}^{tot} \\ Cov_{m,p}^{tot} & Var_p^{tot} \end{bmatrix},$$

where each of the matrix entries is a sum of a term resulting from intrinsic stochasticity and a term resulting from parameter variability (2):

$$\begin{aligned} Var_m^{tot} &= \mu_{Var_m} + \Sigma_{\bar{m},\bar{m}} \\ Var_p^{tot} &= \mu_{Var_p} + \Sigma_{\bar{p},\bar{p}} \\ Cov_{m,p}^{tot} &= \mu_{Cov_{m,p}} + \Sigma_{\bar{m},\bar{p}}. \end{aligned}$$

Correlated extrinsic variability matrices

Covariance matrices constructed from random correlation matrices and the following diagonals (corresponding to parameters $(k_r, k_p, \gamma_r, \gamma_p)$) result in small differences in total protein variability (similar to Figure 12A):

$0.2, 0.01, 0.01, 0.01$
$0.01, 0.2, 0.01, 0.01$
$0.03, 0.2, 0.03, 0.03$
$0.05, 0.2, 0.01, 0.01$
$0.2, 0.05, 0.01, 0.1$
$0.03, 0.2, 0.03, 0.01$
$0.03, 0.2, 0.01, 0.03$

The following diagonals result in large differences in total protein variability (similar to Figure 12C):

$0.05, 0.01, 0.03, 0.05$
$0.01, 0.01, 0.1, 0.1$
$0.01, 0.05, 0.01, 0.05$
$0.05, 0.01, 0.05, 0.01$
$0.03, 0.03, 0.03, 0.03$
$0.03, 0.03, 0.05, 0.01$
$0.03, 0.05, 0.03, 0.03$
$0.01, 0.05, 0.01, 0.03$

Propagating log-normal distributions with the unscented transform

Here we derive how to use the unscented transform to propagate log-normally distributed parameters through a nonlinear function f by using the unscented transform. If X is a random variable from $LogN(\mu, \sigma^2)$, then $Y = ln(X)$ is a random variable from $N(\mu, \sigma^2)$. Propagating X through our original function f results in the same output distribution as propagating Y through a function g, where

$$f(X) = f(e^{ln(X)}) = f(e^Y) = g(Y).$$

Therefore, to propagate $LogN(\mu, \sigma^2)$ through a function f, we can calculate sigma points y_i from $N(\mu, \sigma^2)$, and use the UT to propagate them as $f(e^{y_i})$ for all i and reconstruct them into the sought output distribution.
